# Influence of vascular risk factors and vascular disease on long-term outcome in idiopathic Normal Pressure Hydrocephalus; a Quality Registry based study

**DOI:** 10.1186/2045-8118-12-S1-O16

**Published:** 2015-09-18

**Authors:** Kerstin Andrén, Carsten Wikkelsö, Nina Sundström, Katarina Laurell, Babar Kahlon, Per Hellström, Mats Tullberg

**Affiliations:** 1 Institute of Neuroscience and Physiology, The Sahlgrenska Academy, University of Gothenburg, Sweden; 2 Department of Pharmacology and Clinical Neuroscience, Umeå University, Sweden; 3 Department of Neurosurgery, University Hospital, Lund, Sweden

## Introduction

Studies support a higher proportion of vascular risk factors in iNPH patients than in the general population. Less favorable outcome for patients with, than without, cerebrovascular comorbidity and vascular risk factors has been described, though in smaller studies. We investigate whether this can be confirmed in a large national Quality Registry based study.

## Methods

Pre-and postoperative data were extracted from the Swedish Hydrocephalus Quality Registry (SHQR) on all iNPH patients (n=981) operated 2004-2011 including ordinal scales for gait, balance and continence, Mini Mental State Examination and Modified Rankin Scale (mRS), incidence of hypertension, diabetes, heart disease or stroke. In addition, data were collected from follow-up letters at 2-10 years after surgery: a self-evaluation form describing patients' present function and subjective comparison to their pre-operative condition.

A control population is currently being selected by the bureau Statistics Sweden and data from the Swedish Registry of Cause of Death will be obtained for patients and controls; an ongoing expansion of the project.

## Results

At time of inclusion in the registry n=434 (50%) had hypertension, n=186 (21%) had diabetes, n=119 (14%) were stroke-sufferers and n=229 (26%) had heart disease. The magnitude of change in the different outcome measures 3 months after surgery was not influenced by any of the risk factors investigated.

After 2 and 5 years, the proportion of patients who survived was smaller for patients with heart disease than without heart disease, but similar for the other three vascular factors.

In total, n=623 (64%) of patients returned 925 follow-up letter responses 3.5 ±1.4 (mean, SD) years after surgery; 2 and 5 year data are reported here.

Two and 5 years after surgery, 61.3% and 64.7% respectively, reported themselves still improved. Vascular factors did not influence the subjective reports of improvement.

Univariate analysis 2 years postoperatively for each of the four reported vascular factors showed no increased risk for poor outcome, defined as mRS 3-5 or 6 (deceased). At 5 years postoperatively OR for poor outcome with heart disease at baseline was 3.04 (1.52-6.05 95%CI), p=0.002; stroke OR 2.93 (1.11-7.70 95%CI), p=0.029; diabetes OR 2.43 (1.15-5.17 95%CI), p=0.021; Hypertension n.s.

## Conclusion

Preliminary results from this Quality Registry based study with 981 iNPH patients suggest that co-occurrence of vascular factors do not increase the risk for poor outcome within 2 years from shunt surgery, but at 5 years postoperatively there is an increased risk. For surviving patients, the vascular risk factors have no influence on the proportion reporting themselves still improved 2 and 5 years after surgery.
